# Multiple Roles of Paeoniflorin in Alzheimer's Disease

**DOI:** 10.1155/2022/2464163

**Published:** 2022-04-11

**Authors:** Zeyu Meng, Huize Chen, Chujun Deng, Shengxi Meng

**Affiliations:** ^1^The Second Clinical Medical College, Heilongjiang University of Chinese Medicine, Harbin 150040, China; ^2^Department of Traditional Chinese Medicine, Shanghai Jiao Tong University Affiliated Sixth People's Hospital, Shanghai 200233, China

## Abstract

Alzheimer's disease (AD) is a geriatric disease with the morbidity and mortality continuing to grow, partly due to the aging of the world population. As one of the most common types of primary neurodegenerative dementia, it is mainly due to environmental, epigenetic, immunological, and genetic factors. Paeoniflorin (PF), the main component of paeony extract, plays a more and more important role in the prevention and treatment of AD, including regulating protein, anti-inflammation, antioxidation, and antiapoptosis, protecting glial cells, regulating neurotransmitters and related enzymes and receptors, and inhibiting or activating related signal pathways. This article summarizes the latest researches on the multiple effects and the mechanisms of PF in the treatment to cure AD, providing new insights and research basis for further clinical application of traditional Chinese medicine (TCM) in the treatment of AD.

## 1. Introduction

Alzheimer's disease is a degenerative disease of the central nervous system that occurs in the presenile and elderly. It will show short-term memory degradation, speech repetition, slow response, language function degradation, obvious decline in understanding and expression ability, and so on. The pathological manifestations were diffuse atrophy of cortex, enlargement of ventricle, and widening of sulcus gyrus [1, 2]. *β*-Amyloid protein (A*β*) deposition and hyperphosphorylated tau nerve fiber tangles are typical pathological features of AD [3, 4]. At present, there is no specific therapy for the treatment of Alzheimer's disease; acetylcholinesterase inhibitors are commonly used in clinical treatment and the effectiveness of more treatment methods needs to be further verified.

Along with AD, traditional Chinese medicine (TCM) is also coming into view. The field of TCM shows unique potential and advantages, such as multitarget, multiapproach, and multilevel. In recent years, TCM prescriptions such as Dihuang Yinzi [5], Banxia Baizhu Tianma Tang [6], Danggui Shaoyao San [7], and Kaixinsan [8] have been used to treat AD, while Radix ginseng-*Schisandra chinensis* herb pair [9], *Astragalus Radix*-Ligustri Lucidi Fructus herb pair [10], Danshen-Chuanxiong herb pair [11], Rehmanniae Radix Praeparata-Radix Polygoni Multiflori herb pair [12], Radix Polygoni Multiflori-Acori Tatarinowii Rhizoma herb pair [13],and other traditional Chinese medicine pairs have been used to prevent and treat AD.

The monomer components extracted from TCM have become a new research hotspot because of their high efficiency, low toxicity, strong specificity, multi-target, and other characteristics, which can play a role in the treatment of AD in multiple ways. TCM monomer components are mainly divided into alkaloids, phenylpropanoids, flavonoids, saponins, terpenoids, glycosides, and so on. At present, the studies on the therapeutic mechanism of AD are mainly focused on berberine [14–16], evodiamine [17], icariin [18, 19], forsythoside [20], ferulic acid [21], osthole [22, 23], triptolide [24], resveratrol [25], and rhynchophylline and isorhynchophylline [26]. However, there has been a lack of systematic review and research on the relationship between a component of *Radix Paeoniae* and AD.

Radix Paeoniae Alba is the dried rhizome of Ranunculaceae plant *Paeonia lactiflora Pall*. It is usually picked in summer and autumn and stir-fried or roasted until yellowish in order to be used as medicine. It is recorded in Shennong's Classic of the Materia Medica, firstly. As one of the traditional Chinese herbal medicines, it is bitter, sour in flavor, and slightly cold in nature, which is mainly reflected in the therapeutic effects of astringing yin and stopping sweating, nourishing blood and regulating menstruation, softening the liver and relieving pain, restraining liver Yang, and so on.

**Figure 1:**
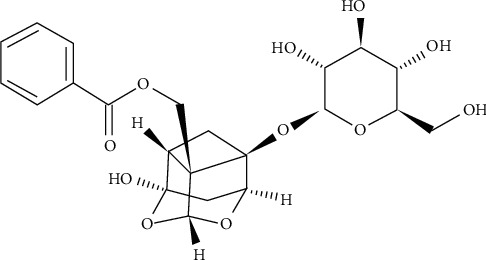
Chemical structure of Paeoniflorin.

Paeoniflorin (PF), one of the major components of Radix Paeoniae extract, has been clinically used in China and other East Asian countries for possession of antidementia properties and the treatment of neurodegenerative disease. Its molecular formula is C_23_H_28_O_11_ (Figure 1). Many studies indicate that PF has the important function of regulating protein, anti-inflammation, antioxidation, antiapoptosis, protecting glial cells, and so on. This review focuses on the latest progress in the role and mechanism of PF in AD and provides a novel perspective on clinical application.

## 2. Regulatory Protein

### 2.1. Reduction of Excessive Deposition of A*β*

The pathological changes of AD include the deposition of insoluble A*β* in extracellular and the accumulation of tau protein in intracellular nerve fiber tangles [27, 28]. AD is a neurodegenerative disease related to age and the toxic form of A*β* peptide. The toxic form of A*β* peptide increases with age, and abnormal folding eventually accumulates to form senile plaque (SP) [29, 30]. PF mediates neuroprotective effects such as reduction of neuroinflammation, reduction of amyloid *β* plaque load, and decreased expression of IL-1*β* and TNF-*α* in a transgenic mouse model of AD by activating adenosine A_1_R [31]. PF is confirmed to inhibit the phosphorylation of NF-*κ*B and increase the protein expression of kappa B-*α* and A*β* degrading enzymes to reduce the excessive deposition of A*β* [32].

Research shows that heat shock protein-16.2 (hsp-16.2) plays a significant role in the clearance of misfolded and unfolded proteins in *Caenorhabditis elegans* and PF can increase the expression of hsp-16.2 in the AD model of *C. elegans* induced by A*β*_1–42_ to clear A*β*, delaying *C. elegans* paralysis caused by the accumulation of A*β*, significantly [33]. On the contrary, PF was thought to treat AD not by removing A*β* and its fibrous plaques but by inhibiting the production of A*β* to reduce the aggregation of A*β* to form fibrous plaques in the experimental rat model [34]. Therefore, the specific mechanism of the influence of PF on A*β* is still controversial and needs to be further explored.

### 2.2. Inhibition of Abnormal Phosphorylation of Tau Protein

**Figure 2:**
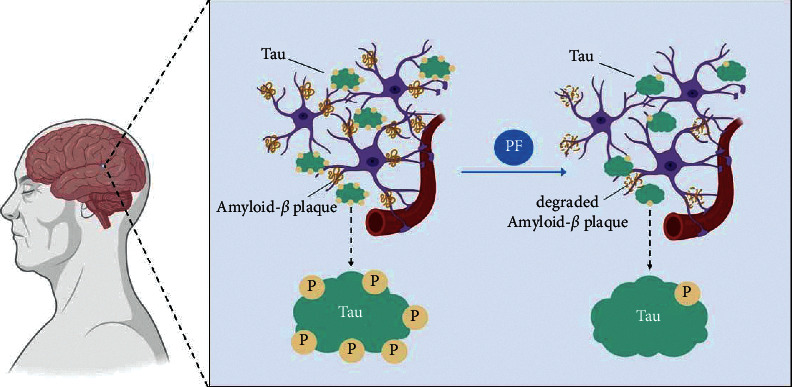
Changes of amyloid-*β* plaque and tau in the treatment of PF.

Tau, as a microtubule-associated protein, is the main neuropathological marker of AD [35]. Neurofibrillary tangle (NFT) is formed by hyperphosphorylated tau accumulation, and extracellular amyloid plaques are composed of A*β* [36]. In addition to participating in the formation of neurofibrillary tangles, the accumulation of hyperphosphorylated tau can also lead to neuronal dysfunction and synaptic damage. It is also one of the reasons why CSF and blood tau phosphorylated at threonine could be used as a biomarker for Alzheimer's disease and for the prediction of cognitive decline [37]. Intracellular MAPT/tau accumulation induced by macrophage/autophagy deficiency is a landmark pathological feature of AD [38], while the autophagy-lysosomal pathway (ALP) is the main pathway for clearance for tau in neurons [39]. Tau can be degraded through the autophagy mTOR pathway, thus relieving the symptoms of AD [38]. The regulation of autophagy can degrade hyperphosphorylated tau to some extent. While PF antagonizes the calpain/Akt/glycogen synthase kinase 3*β* (GSK-3*β*) signal pathway, autophagy is stimulated. And, the phosphorylation/activation of GSK-3*β* is enhanced by autophagy stimulation, resulting in the subsequent hyperphosphorylation of tau [40]. Research showed that IRS-1 and its downstream effector molecules can participate in tau protein hyperphosphorylation. PF pairs prevent tau hyperphosphorylation and protect cognitive impairment by restoring SOCS2/IRS-1 signal pathway [41] (Figure 2 and Table 1).

## 3. Protection of Neurons

### 3.1. Inhibition of Inflammatory Response Associated with TNF-*α* and IL-1*β*

The inflammatory response of the central nervous system (CNS) is considered to be a very complex defense mechanism. Chemokines secreted by CNS cells, such as TNF-*α* and IL-1*β*, which are involved in the mechanism of inflammation, may play a variety of important roles in AD [42–45].

PF exerts neuroprotective effects such as decreased expression of IL-1*β* and tumor necrosis factor-alpha (TNF-*α*) and reduction of neuroinflammation in 5XFAD mice by activating adenosine A_1_R [31]. Research suggested that inflammation-induced overexpression of SOCS2 can lead to cognitive dysfunction. PF can reduce cognitive dysfunction via regulating SOCS2/IRS-1 signal transduction and blocking tau hyperphosphorylation. Meanwhile, Paeoniflorin can decrease the contents of TNF-*α* and IL-1*β* in the hippocampus to reduce inflammation [42]. The involvement of the translocator protein 18 kDa (TSPO) is a biomarker of neuroinflammation in vivo [46, 47]. The neuroprotective effect of PF on neurodegenerative diseases such as AD may be mediated by TSPO and its downstream neurosteroids [48]. PF is confirmed to inhibit TNF-*α* and IL-1*β* and the activity of NLRP3 inflammasome to relieve inflammatory reaction [49]. PF can downregulate the protein expression of inducible nitric oxide synthase and cyclooxygenase-2, inhibit the NO production of C_6_ glial cells induced by A*β*_25–35_, and has a protective effect on A*β*-mediated neuroinflammation [32]. The increase of chemokine level can make more microglia aggregate to A*β*. PF can inhibit the secretion of proinflammatory mediators IL-1*β*, IL-6, and TNF-*α* and chemokine CCL2 and CXCL1 by microglia induced by A*β*_1–42_ and then treat AD [50].

### 3.2. Inhibition of Inflammatory Response Associated with DHA Metabolic Signal Pathway

**Figure 3:**
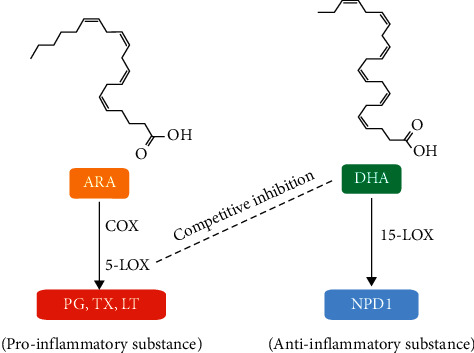
DHA metabolic signal pathway related to Paeoniflorin and Danggui Shaoyao San. The solid line represents promotion and the dashed line represents inhibition.

Docosahexaenoic acid (DHA) is an essential high unsaturated fatty acid for brain nutrition, which is beneficial to the growth and development of brain nerve conduction and synapse [51]. Arachidonic acid (ARA) produces PG (prostaglandin), LT (leukotriene), or TX (thromboxane) under the action of COX and 5-LOX. These three active substances have obvious proinflammatory effects. In addition, DHA can produce NPD1 with anti-inflammatory activity and neuroprotective effect under the action of 15-LOX. Interestingly, although DHA and ARA are the main unsaturated fatty acids in the brain, DHA can competitively inhibit the synthesis of LT by ARA, such as LTA4, LTB4, TXB2, and other proinflammatory substances, and then play their own anti-inflammatory effects. On the one hand, Danggui Shaoyao San upregulated the expression of 15-LOX in the DHA metabolic pathway to increase the content of NPD1 in the brain; on the other hand, it downregulated the metabolic enzymes COX family and 5-LOX, thus reducing the proinflammatory activity of PG and LTA4, LTB4, and TXB2 (Figure 3). The therapeutic effect of Danggui Shaoyao San on AD is related to PF [52], and its deeper mechanism needs to be further verified.

### 3.3. Antioxidant Stress Injury

Oxidative stress and mitochondrial function have long been considered to play a key role in neurodegenerative diseases including AD [53, 54]. Treatment with PF significantly alleviates the degree of oxidative stress as exhibited by the reduction of glutathione (GSH) and superoxide dismutase (SOD) activity. Treatment with PF also significantly alleviates mitochondrial dysfunction as exhibited by increasing cytochrome c oxidase activity and ATP synthesis. PF can improve cognitive impairment and the defect of insulin signal transduction by upregulating the expression of p-PI3K and p-Akt protein and downregulating the expression of p-IRS-1 protein [55].

Reactive oxygen species (ROS) is a natural by-product containing oxygen, which plays a vital role in cell signal transduction. When the redox-active metal ions in the body combine with A*β*, it can catalyze the production of ROS. The increase of ROS level may cause serious damage to the A*β* peptide itself and other proteins [56–58]. Danggui Shaoyao San (DSS) can significantly reduce the contents of ROS and increase the activities of GSH and SOD in the hippocampus and cortex of APP/PS1 mice, which plays a role in improving the state of oxidative stress in the brain, which is closely related to PF [52].

### 3.4. Reduction of Apoptosis

Apoptosis is widely known as programmed cell death that can be triggered by both intracellular and extracellular signals, which can effectively remove damaged cells, such as DNA damaged cells and mitochondrial oxidative phosphorylation damaged cells [59, 60]. MKK4 is a kind of stress-activated protein kinases (SAPKs), which can activate JNK1 and JNK2 at the same time, regulate transcription factors, and finally participate in the regulation of other cellular processes, such as proliferation, differentiation, and suppression of metastases [61, 62].

Bcl-2 family proteins are the main regulators of modulating cell death and survival, which are divided into two categories: the antiapoptotic genes and proapoptotic genes. Bcl-2-associated *X* protein (Bax) is an important member of the Bcl-2 family, which can activate a series of downstream genes and induce apoptosis by antagonizing Bcl-2 [63]. Meanwhile, the caspase family and Bcl-2 family play significant roles in the process of apoptosis [64]. PF is proved to improve the cognitive impairment of AD transgenic mice by increasing the Bcl-2/Bax ratio, reducing caspase-3 activity, and inhibiting apoptosis [65]. PF can modulate the Bcl-2/Bax ratio and downregulate cleaved-caspase-3 levels via inhibition of MKK4-JNK signaling pathway to suppress TBTC-induced apoptosis and damage on neurons and treat eventually neurodegenerative diseases such as AD [66]. This is listed in more detail in Table 2.

## 4. Protection of Neuroglial Cells

### 4.1. Inhibition of Activation of Microglia

The central nervous system (CNS) consists of two broad categories of cells: neurons and neuroglial cells. Microglia are the main resident immune cells of the brain and the innate immune cells of the CNS of myeloid origin, which together with oligodendrocytes, astrocytes, and ependymal cells form the neuroglial cells [67, 68]. Studies have shown that microglia account for 5–12% of central nervous system cells [69]. The dominant view is that microglia mainly originate from bone marrow hematopoietic stem cells in mesoderm [68]. It is also believed that microglia originated from erythromyeloid progenitor cells in the embryonic yolk sac [70]. Its main function is to eliminate microbes, phagocytize necrotic or apoptotic cells, protein aggregates, redundant synapses, and other antigens and particulate that may endanger the CNS [71]. Some study results showed that PF can reduce the overexpression of microglia marker Iba1 in the hippocampus induced by lipopolysaccharides (LPS) and inhibit the activation of microglia. It probably has a neuroprotective effect by inhibiting the activation of hippocampal microglia and activating the neuronal FGF-2/FGFR1 signal pathway [72]. Although there is no conclusive evidence that PF treats AD by protecting microglia, the treatment of PF plays a key role in the inhibition of the activation of microglia [49].

### 4.2. Protection of Astrocyte

Astrocytes are a kind of neuroglial cells, which play essential roles in supplying energy to neurons, modulating local blood flow, neural circuit function, synaptic plasticity, and development [73, 74]. This study has shown that neurovascular injury during the onset of AD would cause astrocyte atrophy, which, in turn, promotes the deterioration of AD [75]. Thus, as one of the early features of AD, the reactivity of astrocytes is expected to become an important target for preclinical diagnosis and treatment [76]. PF can activate adenosine A_1_R and further alleviate astrocyte activation and neuroinflammation in 5XFAD mice to improve the symptoms of AD [31]. Research showed that PF protects astrocytes by participating in the biosynthesis of TSPO and neurosteroids and then plays a therapeutic role in neurodegenerative diseases such as AD [47]. PF can inhibit the release of microglial chemokine CCL2 and CXCL1 stimulated by A*β*_1–42_, reduce the chemotaxis of microglia, and then treat AD [49].

### 4.3. Protection of Oligodendrocytes

Neuroimaging studies show that white matter degeneration and demyelination may also be important pathophysiological features of AD, and the formation of myelin is closely related to oligodendrocytes [77]. Considerable research implicated that myelin destruction and oligodendrocyte dysfunction may cause reduction of excessive deposition of A*β* through neuroinflammation. Oligodendrocytes may be a new breakthrough in the prevention and treatment of AD [78]. Shenzhiling oral solution can protect oligodendrocytes by downregulating the acetylation level of histone H3 and the level of MBP gene by epigenetic regulation. The protective effect of Shenzhiling oral solution on oligodendrocytes is related to PF [79] (Table 3).

## 5. Regulation of Neurotransmitters-Related Enzymes and Receptors

### 5.1. Inhibition of Ca^2+^-Related Proteases

Calpain is a kind of calcium-dependent cysteine protease, which is mainly divided into three types: calpain1 (u-calpain), calpain2 (m-calpain), and calpain3 (p94). Studies have shown that PF may inhibit u-calpain by reducing the concentration of calcium in SH-SY5Y cells induced by okadaic acid (OA) and eventually reversed tau hyperphosphorylation [40].

### 5.2. Regulation of Acetylcholine-Related Enzymes

The cholinergic system of the brain is closely related to age-related cognitive decline. Studies indicate that the gradual loss of cholinergic innervation in the margin and neocortex is one of the reasons for the formation of AD. The loss of innervation is closely related to the synthesis and hydrolysis of acetylcholine involved in choline acetyltransferase (ChAT) and acetylcholinesterase (AChE). It has been found that the increase of AChE in the brain of patients with AD can promote the excessive deposition of A*β*, while the decrease of ChAT transport can lead to the aggravation of dementia symptoms [80–83]. PF could decrease the activity of AChE and increase the activity of ChAT in the brain of A*β*_1–42_-induced AD rats and restore the cholinergic system and innervation to normal [84].

### 5.3. Activation of Adenosine Receptor

Adenosine is widely distributed in the CNS and plays a neuroprotective role. Most adenosine functions are mediated by receptors, including A1, A2A, A2B, and A3 receptors (A1R, A2AR, A2BR, and A3R). They can control the release of neurotransmitters including glutamate and acetylcholine, which are involved in the learning and cognitive process, and affect these adenosine receptors, which may change the process of AD to some extent. [85–87]. The neuroprotective effect of PF is mediated by the activation of adenosine A_1_R. It can significantly reduce the load of A*β* plaque in the brain of mice (Table 4).

## 6. Inhibition/Activation of Related Signal Pathway

### 6.1. Inhibition of MAPK Pathway

Mitogen-activated protein kinase (MAPK) family is a kind of serine/threonine protein kinase, which is a group of major signal molecules in the process of signal transduction, and its activation is the final step of intracellular phosphorylation cascade. P38 is the most significant member of MAPK family to control inflammatory response [88, 89]. PF showed therapeutic activities and neuroprotective effect against AD through suppression of the p38 MAPK pathway, alleviating bupivacaine-induced neurotoxicity in neural cells [90]. PF is confirmed to improve the cognitive impairment of AD mice by downregulating the expression of p-p38MAPK and reducing caspase-3 activity and inflammatory reaction [65].

### 6.2. Inhibition of GSK-3*β* and NF-*κ*B Pathway

NF-*κ*B protein is a homologous/heterodimer formed by p65 and p50, which is related to synaptic plasticity and memory. GSK-3*β* is a serine/threonine kinase, which is ubiquitous in mammalian eukaryotic cells. It can act on many signal proteins, structural proteins, and transcription factors and regulate cell apoptosis, differentiation, and proliferation. Although the role of PF in the treatment of AD through GSK-3*β* and NF-*κ*B pathway is not completely clear, current studies have shown that PF is likely to inhibit the activation of GSK-3*β* and NF-*κ*B pathway [49], which suppressed the production of IL-6, IL-1*β*, and tumor necrosis factor-alpha (TNF-*α*) [32]. The relevant mechanism needs to be further verified.

### 6.3. Activation of PI3K/Akt/mTOR Pathway

**Figure 4:**
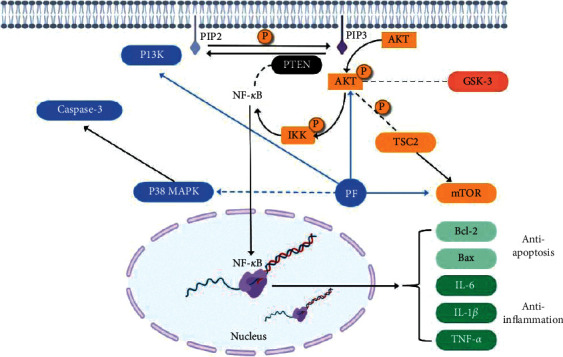
Some possible partial signal pathways related to inhibition/activation of Paeoniflorin. The solid line represents promotion and the dashed line represents inhibition.

This study proves that PI3K is an intracellular phosphatidylinositol kinase composed of p85 and p110. Thus, Akt is a protein serine/threonine kinase that acts on cell survival and apoptosis. The mammalian target of rapamycin (mTOR) is an important regulator of cell growth and proliferation. Shenzhiling oral solution may protect myelin sheath and treat AD by upregulating the expression of PI3K, Akt, and mTOR [79] and increasing their phosphorylation. The protective effect of Shenzhiling oral solution is closely related to PF [91] (Figure 4 and Table 5).

## 7. Summary and Prospect

PF plays a more and more important role in AD, including regulating protein, anti-inflammation, and antioxidation, protecting glial cells and antiapoptosis, regulating neurotransmitters-related enzymes and receptors, activating or inhibiting related signal pathways, and so on. Although the current research on the mechanism of PF in the treatment of AD is very extensive, the vast majority of them are focused on animal experiments and cell experiments, have a lack of large samples of clinical trials and clinical observation, and have not studied clinical dose-effect relationship. In this regard, researchers need to conduct large-scale, randomized, controlled, double-blind clinical trials to further demonstrate the conclusions of animal experiments and cell experiments, in order to accurately explore the potential clinical role and mechanism of PF in AD.

In recent years, the research focus on the mechanism of the action of PF on AD ranges from neurons to the type of neuroglial cells such as microglia astrocytes oligodendrocytes. Some studies have made a new interpretation of the mechanism of PF to AD from the perspective of lipid metabolism and epigenetics. The further deepening of the research also indicates that researchers have a deeper understanding of the relationship between PF and AD. PF therapy is expected to become a new method and new idea for the prevention and treatment of AD, which will benefit more AD patients.

However, there is still a huge research space in this field. Whether there is a potential relationship between these mechanisms and mechanisms and whether different mechanisms are different forms of expression of the body will be further breakthroughs in future research.

## Figures and Tables

**Table 1:** The mechanism of regulating proteins in the treatment of AD with Paeoniflorin.

Included studies	Year	Animal experiment		Clinical trial	Possible mechanisms (signaling pathway)
		Animal/cell	Disease model		
Kong et al. [31]	2020	5XFAD mice	A novel transgenic mouse model of AD	—	Reduction of A*β* plaque load and reduction of neuroinflammation by activating adenosine A_1_R
Cho et al. [32]	2020	C_6_ glial cells were treated with PF and A*β*_25–35_	An AD cellular model	—	Inhibiting the phosphorylation of NF-*κ*B and increasing the protein expression of KappaB-*α* and A*β* degrading enzymes
Ai et al. [33]	2017	*Caenorhabditis elegans* induced by A*β*_1–42_	A nematode model of AD	—	Delaying significantly *C. elegans* paralysis caused by toxic A*β* oligomer by increasing the expression of hsp-16.2
Zhou et al. [34]	2016	5XFAD mice	A novel transgenic mouse model of AD	—	Inhibiting the production of A*β* to reduce the aggregation of A*β* to form fibrous plaques
Ma et al. [40]	2018	OA-treated SH-SY5Y cells	An AD cellular model	—	Antagonizing the calpain/Akt/GSK-3*β*-related signal pathway and stimulating autophagy
Sun et al. [41]	2017	Male Sprague–Dawley (SD) rats	A diabetic rat model with cognitive impairment	—	Preventing tau hyperphosphorylation via recovering SOCS2/IRS-1 signaling

**Table 2:** The mechanism of protection of neurons in the treatment of AD with Paeoniflorin.

Included studies	Year	Animal experiment		Clinical trial	Possible mechanisms (signaling pathway)
		Animal/cell	Disease model		
Kong et al. [31]	2020	5XFAD mice	A novel transgenic mouse model of AD	—	Decreased expression of IL-1 *β* and TNF-*α* and reduction of neuroinflammation and by activating adenosine A_1_R
Sun et al. [41]	2017	Male Sprague–Dawley (SD) rats	A diabetic rat model with cognitive impairment	—	Preventing tau hyperphosphorylation via recovering SOCS2/IRS-1 signaling and decreasing the contents of TNF-*α* and IL-1*β* in the hippocampus
Qiu et al. [48]	2018	Rat astrocyte cells	A cellular model of neurodegenerative diseases	—	The cytoprotection mediated by TSPO and neurosteroids biosynthesis
Cho et al. [32]	2020	C_6_ glial cells were treated with PF and A*β*_25–35_	An AD cellular model	—	Inhibiting the NO production of C_6_ glial cells
Liu and Wang [50]	2017	Primary microglia of SD rats	An AD cellular model	—	Inhibiting the secretion of proinflammatory mediators IL-1 *β*, IL-6, TNF-*α*, and chemokine CCL2 and CXCL1
Gu et al. [65]	2016	C_57_BL/6 × DBA/2 mice	A transgenic mouse model of AD	—	Increasing Bcl-2/Bax ratio, reducing caspase-3 activity, and inhibiting apoptosis
Wang [52]	2018	APP/PS1 mice	A mouse model of AD	—	Decreasing the content of ROS, increasing the content of GSH and SOD, upregulating the expression of 15-LOX, increasing the content of NPD1, and reducing the formation of PG, TXB2, and LTB4
Cong et al. [66]	2019	TBTC-induced hypothalamic neurons from neonatal rats	An AD cellular model	—	Inhibition of MKK4-JNK signaling pathway, modulation of the Bcl-2/Bax ratio, and downregulation of cleaved-caspase-3 levels
Hu et al. [49]	2018	C57BL/6J mice established by intraplantar injection of CFA	An inflammatory model	—	Inhibition of TNF-*α* and IL-1*β* and the activity of NLRP3 inflammasome to relieve the inflammatory reaction

**Table 3:** The mechanism of protection of neuroglial cells in the treatment of AD with Paeoniflorin.

Included studies	Year	Animal experiment		Clinical trial	Possible mechanisms (signaling pathway)
		Animal/cell	Disease model		
Cheng et al. [72]	2021	ICR mice	A mouse model of neuroinflammation	—	Inhibiting the activation of hippocampal microglia and activating neuronal FGF-2/FGFR1 signal pathway
Liu and Wang [50]	2017	Primary microglia of SD rats	An AD cellular model	—	Inhibiting the release of microglial chemokine CCL2 and CXCL1 stimulated by A*β*_1–42_ and reducing the chemotaxis of microglia
Kong et al. [31]	2020	5XFAD mice	A novel transgenic mouse model of AD	—	Decreased expression of IL-1 *β* and TNF-*α* and reduction of neuroinflammation by activating adenosine A_1_R
Qiu et al. [48]	2018	Rat astrocyte cells	A cellular model of neurodegenerative diseases	—	Protecting astrocytes by participating in the biosynthesis of TSPO and neurosteroids
Liu et al. [79]	2020	APPswe/PS1dE9-double transgenic model mice	A transgenic mouse model of AD	—	Downregulation of histone H3-acetylated MBP gene level to protect oligodendrocytes

**Table 4:** The mechanism of regulation of neurotransmitters-related enzymes and receptors in the treatment of AD with Paeoniflorin.

Included studies	Year	Animal experiment		Clinical trial	Possible mechanisms (signaling pathway)
		Animal/cell	Disease model		
Ma et al. [40]	2018	OA-treated SH-SY5Y cells	An AD cellular model	—	Activate *u*-calpain resulting in the phosphorylation/activation of Ak and GSK-3*β* and subsequently the hyperphosphorylation of tau
Lan et al. [84]	2013	A*β*_1–42_-induced rats	A novel mouse model of AD	—	Decreasing the activity of AChE and increasing the activity of ChAT
Kong et al. [31]	2020	5XFAD mice	A novel transgenic mouse model of AD	—	Decreased expression of IL-1 *β* and TNF-*α* and reduction of neuroinflammation by activating adenosine A_1_R

**Table 5:** The mechanism of individual signal pathway in the treatment of AD with Paeoniflorin.

Included studies	Year	Animal experiment		Clinical trial	Possible mechanisms (signaling pathway)
		Animal/cell	Disease model		
Moreno-Cugnon et al. [89]	2018	Bupivacaine-induced SH-SY5Y cells	An AD cellular model	—	Suppression of the p38 MAPK pathway
Katsouri et al. [73]	2018	C57BL/6J mice established by intraplantar injection of CFA	An inflammatory model	—	Inhibition of NF-*κ*B pathway and the activation of GSK-3*β*
Gu et al. [65]	2016	C57BL/6 × DBA/2 mice	A transgenic mouse model of AD	—	Downregulating p-p38 MAPK expression
Cho et al. [32]	2020	C_6_ glial cells were treated with PF and A*β*_25–35_	An AD cellular model	—	Inhibiting the activation of GSK-3 and NF-*κ*B pathway
Liu [79]	2020	APPswe/PS1dE9 double transgenic model mice	A transgenic mouse model of AD	—	Enhancing PI3K/Akt-mTOR signal pathway
Chen et al. [90]	2019	Rat oligodendrocyte OLN-93 cells injured by STZ	An AD-like cell model in vitro	—	Enhancing PI3K/Akt-mTOR signal pathway

## Data Availability

The data used in the current study are included within this article.
